# The determinants of dietary diversity and nutrition: ethnonutrition knowledge of local people in the East Usambara Mountains, Tanzania

**DOI:** 10.1186/s13002-017-0150-2

**Published:** 2017-04-27

**Authors:** Bronwen Powell, Rachel Bezner Kerr, Sera L. Young, Timothy Johns

**Affiliations:** 1 Department of Geography and African Studies Program, Pennsylvania State University, State College, PA USA; 2 Center for International Forestry Research, Bogor, Indonesia; 3 Department of Development Sociology, Cornell University, Ithaca, NY USA; 4 Department of Anthropology, Institute for Policy Research, Northwestern University, Evanston, Illinois USA; 5 School of Dietetics and Human Nutrition, McGill University, Montreal, Canada

**Keywords:** Agrobiodiversity, Local knowledge, Dietary behaviour, Dietary diversity, Landscape diversity

## Abstract

**Background:**

Diet and nutrition-related behaviours are embedded in cultural and environmental contexts: adoption of new knowledge depends on how easily it can be integrated into existing knowledge systems. As dietary diversity promotion becomes an increasingly common component of nutrition education, understanding local nutrition knowledge systems and local concepts about dietary diversity is essential to formulate efficient messages.

**Methods:**

This paper draws on in-depth qualitative ethnographic research conducted in small-scale agricultural communities in Tanzania. Data were collected using interviews, focus group discussions and participant observation in the East Usambara Mountains, an area that is home primarily to the Shambaa and Bondei ethnic groups, but has a long history of ethnic diversity and ethnic intermixing.

**Results:**

The data showed a high degree of consensus among participants who reported that dietary diversity is important because it maintains and enhances appetite across days, months and seasons. Local people reported that sufficient cash resources, agrobiodiversity, heterogeneity within the landscape, and livelihood diversity all supported their ability to consume a varied diet and achieve good nutritional status. Other variables affecting diet and dietary diversity included seasonality, household size, and gender.

**Conclusions:**

The results suggest that dietary diversity was perceived as something all people, both rich and poor, could achieve. There was significant overlap between local and scientific understandings of dietary diversity, suggesting that novel information on the importance of dietary diversity promoted through education will likely be easily integrated into the existing knowledge systems.

**Electronic supplementary material:**

The online version of this article (doi:10.1186/s13002-017-0150-2) contains supplementary material, which is available to authorized users.

## Background

“My grandfather used to advise me that if you want to have a good life, a good life is not to have money, it is about food which ensures that you will not be troubled… If you want a good diet you must have foods for changing your diet.” Ramadhani Juma, Tongwe village

Malnutrition, including undernutrition, micronutrient deficiency and overnutrition, remains one of the biggest challenges to global development [1–3]. Ensuring healthy diets, along with reducing infectious disease, is the foundation of long-term and sustainable strategies for overcoming global malnutrition [4–6]. However, dietary practices are determined by a complicated mix of biology, knowledge, skills, social-cultural factors (such as identity and beliefs), psychological factors (emotion, motivation, goals, memory, and attention), environmental context, and resources [7–9]. There is growing recognition of the important role of structural, environmental, cultural, social and psychological factors in dietary behaviour [10]. Krumeich et al. [11] note that health decisions are often shaped by factors such as social and cultural context, that are beyond the control of the individual, and that healthful behaviour change is not simply a matter of convincing people to act in a more rational manner.

While traditional diets are often quite healthy [12], social, cultural and economic change in many places has led to dietary transitions associated with decreasing quality of diets [13]. In most contexts, intervention helps to reverse, mitigate or prevent the negative impact of nutrition transitions and the impact of outside drivers of dietary quality. The capacity of a nutrition education program is dependent on the quality of the education program, the acceptability of the message, and local understandings of a particular issue [14]. Important innovation and progress in nutrition education has drawn on psychology and behaviour change theories [15], as well as social marketing and innovative approaches such as inter-generational education [16–21]; however, there is still much room for improvement.

In the face of the underperformance of nutrition education programs, anthropological and ethnomedical perspectives on knowledge systems and learning (also called knowledge exchange or knowledge transmission) may offer novel insight to achieve more efficient exchange and transmission of health and nutrition-related knowledge. This is likely to be particularly true in developing countries and other settings where the knowledge systems of local peoples may be significantly different from scientific knowledge systems.

Anthropological studies of local knowledge systems emphasize the dynamic nature of knowledge and focus on syncretic combinations of local knowledge and other types of knowledge, as well as acknowledging areas of contention [22, 23]. Anthropologists insist that knowledge is fluid; they seek to understand how knowledge changes and what factors mediate that process [24, 25]. When culture is defined as ‘shared knowledge’ [26–30], knowledge, like culture, can be viewed as adaptive: “It seems likely that the range of diversity in individual versions of the ‘common’ culture is not simply a social imperfection, but an adaptive necessity: a crucial resource that can be drawn on and selected from in cultural change” [31], p.88). These approaches to knowledge would suggest that local knowledge is often highly functional, ensuring individual and community well-being [32, 33]. In his review ‘An Anthropology of Knowledge,’ Barth [29] noted: “We all live lives full of raw and unexpected events, and we can grasp them only if we can interpret them—cast them in terms of our knowledge”.

Worsley [34] noted the need to pay careful attention to how knowledge frameworks are built and the ways nutrition knowledge is learnt. People are more likely to maintain healthy behaviours or adopt new ideas or behaviours if they see them as meeting their own needs and aspirations [35]. Examining variations in local knowledge allows for an understanding of the degree of consensus between people from the same cultural group [28], and facilitates the examination of which forms of existing local knowledge are better aligned with scientific ideas of health-positive behaviour. In anthropological literature, knowledge of a culture or society is often referred to as *emic* and outside or scientific knowledge as *etic.* Herein *local knowledge* is defined as that held by local people (this term is synonymous with *traditional knowledge*, which we have chosen not to use because of the implied dichotomy between *traditional* and *modern*) [36].

To our knowledge there have been very few efforts anywhere to examine local knowledge of dietary diversity and nutrition [37]. This research therefore examines local (ethno-) nutrition knowledge in the East Usambara Mountains, Tanzania. The material presented focuses on dietary diversity, including local perceptions about its role in health and nutrition as well as factors that mediate local people’s ability to achieve and maintain a diverse diet. This research represents an important first effort to understand if and how nutrition education messages promoting dietary diversity are aligned with existing knowledge schemes. The results have important implications for understanding if, why, and how efficiently, dietary diversification messages promoted through nutrition education can support good dietary behaviour.

## Methods

### Study site: the East Usambara Mountains, Tanzania

The East Usambara Mountains lie 40 km inland from the port city of Tanga. Human population density in the region is now 61.3 people per square kilometre, with an annual growth rate of 2.4% [38]. The mountains are the home of the Shambaa and Bondei, and the surrounding lowlands are home to the Zigua ethnic group. The area was historically culturally diverse, even before immigration to the area for wage labour opportunities in the tea and timber industries [39, 40]. The political history of Tanzania has ensured that more than 90% of Tanzanians speak Swahili, the national language. In addition to being the *lingua franca*, Swahili is increasingly used in the home, especially in culturally diverse areas such as this site.

Local livelihoods are based on small-scale farming, supplemented with cash crops, wage labour, small business and animal husbandry. Local diets in the East Usambaras are based mainly on maize (*Zea mays* L.) (most commonly prepared as *ugali,* maize flour cooked into a hard porridge), banana (*Musa* spp.), cassava (*Manihot esculenta* Crantz.), beans (*Phaseolus vulgaris* L. and others) and dry fish (such as *dagaa,* small dried fresh water fish, including *Rastrineobola argentea*). Malnutrition, especially micronutrient deficiencies (e.g., vitamin A and iron), remains a problem in the East Usambaras and in Tanzania in general [41].

The East Usambara Mountains were chosen as the field site for this research because the area is known for its high dietary diversity, largely obtained through subsistence activities [39, 42–44]. This area also provides an interesting setting for the study of local food and nutrition knowledge because the high cultural diversity, combined with long history of a shared language use, create the possibility for both high and low cultural consensus.

### Data collection and analysis

Qualitative data collection took place between September 2008 and November 2009 and included focus groups and one-on-one discussions with over 120 people in six villages (Kiwanda, Tongwe, Bombani, Kwatango, Shambangeda and Misalai) [45]. The majority of material herein comes from 15 case study households (*N* = 28 people) that were selected for in-depth qualitative work from a larger sample of 275 households who participated in a household survey to assess diet in relation to biodiversity [46–48]. These 15 case study households were purposefully selected to achieve a range from those with poor diets to those with good diets (including high and low dietary diversity), as well as households with varying livelihood strategies and socioeconomic status. In each household, ethnographic work was supported with participant observation, in-depth interviews and life histories of adult members. Data collection was framed within an EcoHealth framework [49] aimed at understanding people’s perceptions of their diets, the environmental constraints on their diets, and the social and cultural variables that mediate their ability to maintain their preferred diet.

Research underwent ethics approval at McGill University (IRB #916-0708) and the National Ethics Board in Tanzania (COSTECH) and research agreements were signed with village governments. Informed consent was obtained verbally from adults and guardians of children, was recorded by an enumerator, and, confirmed by the lead researcher (BP) prior to participation in the study. No payment was offered to the 15 case study households for their participation in the qualitative work. During consent, all participants requested that they be identified by name when their stories and words were published. Interviews were conducted in Swahili by BP, with support from a translator (Shundi Ndoe). The life story and history of the adults in each household were collected and research topics discussed through in-depth interviews drawing on an interview schedule. Interviews were transcribed and then translated from Swahili to English by a different research assistant (Ruth Adeka and Sylvester Aura), after removal of in-text English.

Text analysis and coding were conducted manually by BP following methods laid out by Bernard [45]. The final transcribed and translated text, including the English and Swahili, was just under 200,000 words. BP read the text multiple times to identify and code descriptions of why dietary diversity was “important” and “drivers (what a household needs to ensure/support)” of dietary diversity. An initial exhaustive list of codes for “importance” and “drivers” was compiled so that all relevant material was included under at least one code. Codes were then grouped into categories. Finally, the all of the text for each category was collected into one place for final analysis of the relative importance (how frequently a given topic was discussed/percent of households in which it was discussed) and identification of themes (what Saldaña calls Pattern and Focused coding [50]).

The results from the quantitative research and a draft of this paper have been returned to the communities and local government officials (two info briefs in Swahili, Additional files 1 and 2). Participants cited herein have had a chance to review the paper (in English, with a translator present to answer questions) and their quotations (in Swahili) and provide comments and corrections. None of the informants requested changes.

## Results

### Local knowledge on the importance of dietary diversity

Local people were very comfortable with the concept of dietary diversity: “eating different types of food” or “changing the diet/foods” (described in Swahili as “*kubadilisha mlo/vyakula*” or “*kukula aina aina ya vyakula mbalimbali*”, among others). Although dietary diversity was one of the focus topics of the research, it frequently came up spontaneously, even before exposure to the research questions. For example, in group discussions on diet, nutrition and well-being in the communities, in which village leaders were asked to rank the households of the village in terms of diet quality, nutrition and health, dietary diversity was an important aspect of how the diets and health of villagers was assessed. Arguments that a household belonged in a higher or lower group, because they had higher or lower dietary diversity, were presented in multiple villages.

Virtually all participants reported that dietary diversity is important because it maintains and enhances appetite. In Tongwe village, Beatrice Akida, who is a single mother and a kindergarten teacher explained:

“The benefit [of changing your diet] is that food should not bore you so that you don’t lose your appetite for eating. Because with one food, many people lose their appetite. That’s why human beings need to change food. Children get an appetite if today you have cooked cassava *ugali*, tomorrow let it be cassava *ugali* with good *mlenda* (*Corchorus* spp.). So tomorrow if you change to *ugali* [of maize] and beans it will be better than eating *ugali* and beans [for many days in a row]. [If you do not change] you will discover the children saying that they are not going to eat, they go to play outside, yet they are hungry.”

Similarly, Saidi Kombo, a well-educated government employee in Misalai village told us:

“If you eat *dagaa* (small dried whitebait fish) today, tomorrow *dagaa*, yes you eat, but you are tired, you think: ‘Now this is how it will be every day? eating *dagaa*?’....it will become boring. You won’t get any pleasure [from eating], you won’t have any appetite. That is why you need to frequently change. You eat *mchicha* (*Amaranthus* spp.) today, tomorrow you eat *kishone nguo* (*Bidens pilosa* L.) the day after maybe you eat *mchunga* (*Launaea cornuta* (Hochst. ex Oliv. & Hiern) C.Jeffrey)… But if you eat only one vegetable (or side dish) every day and *ugali* (stiff porridge) as your staple food every day, if you eat like this, only *ugali* and *mchicha* every day, you won’t have any appetite to eat… If a mama cooks the same vegetable all the time [her family] won’t eat enough. Because children will eat only a little bit and leave the rest.”

Of course on the other hand, preference for diverse foods could motivate people to seek out a more diversified diet.

The importance of a varied diet for improved appetite held across various timeframes: from meal to meal, day to day and season to season. The importance of dietary diversity pertained not only to the diet in general, but also across food groups, including carbohydrate staples, side dishes and fruit (i.e., one should consume different types of vegetables or staples). The importance of diversity for appetite and adequate intake also applied to varieties of a single crop – an indication that local people perceived dietary diversity as intimately linked to agrobiodiversity (cf. “Factors Enhancing and Limiting Dietary Diversity” section, below).

Virtually all participants across a range in gender, age, social and economic status were comfortable discussing the concept of dietary diversity. This suggests that this concept was a salient part of local nutrition knowledge, and, more importantly, that there was a high degree of consensus among individuals across different groups. Although appetite was overwhelmingly the first, most important benefit of dietary diversity discussed by local people, the importance of having different foods in the diet was also linked to the fact that “every food has its own importance”. For example Mathias Martin of Kwatango village noted: “The benefit [of having many different foods] is because everything has got its own value..... Every food crop has got its own value. We eat fruits because every fruit like pineapple (*Ananas comosus* L) helps the blood (*inasaida damu*).”

Mention of vitamins or nutrients as a benefit of dietary diversity was uncommon and only occurred with more educated participants. In virtually all interviews, the concept that vitamins are one of the benefits of dietary diversity was mentioned only after their importance for appetite had been discussed. Indeed, many participants did not report dietary diversity as having any benefit beyond its value for enhanced appetite, and those who did gave vague descriptions of additional benefits.

### Factors enhancing and limiting dietary diversity

#### Agriculture and agrobiodiversity

When discussing factors needed to achieve and maintain a diverse diet, agrobiodiversity,^1^ as well as engaging in agricultural activities in general (maintaining agricultural activities even when there is an alternative source of income), were some of the most commonly mentioned factors. Links between dietary diversity and agrobiodiversity came out as a clear category or theme in 13 out of the 15 case study households. Although local people tended to blur the line between factors affecting dietary diversity and those related to having (enough) food in general, agrobiodiversity seemed to be especially important for ensuring diversity of vegetables and fruit. Zaina Housseni in Tongwe village explained that her family’s diet was better than her neighbours “because, I don’t know, here in the village [other people’s] side dish is *dagaa* unless you have your own garden. I have my own garden with *mchicha* (*Amaranthus* spp.).”

Wealth and available cash were reported to increase dietary diversity directly, as well as indirectly through an influence on agrobiodiversity. Engaging in agriculture (even when pursuing other sources of income) and maintenance of agrobiodiversity were seen as an important strategy for overcoming seasonal variation and food insecurity/hunger, as well as maintaining dietary diversity on both a short- and a long-term basis. Benjamin Njiku, in Shambangeda village noted:

“[Having many varieties of banana] helps us because each variety has a different taste. Also they ripen [at different times].... it allows me to have bananas all the time, each time a different variety. If one variety fails, there is another variety that continues to grow. Also… the time to cook [some varieties] is short, and this is helpful. You can cook quickly, eat quickly. Other [varieties] are a little bit hard and they need a little bit longer.”

The importance of agrobiodiversity included not only the diversity of crops in the field, but also access to different types of fields and fields in diverse locations with different ecological characteristics (i.e., land-use diversity or landscape heterogeneity). Although not well articulated by many, local farmers alluded to the fact that maintaining multiple land uses on their farms helps ensure their food security and dietary diversity. By maintaining different land use types, farmers reported they were able to increase their crop diversity (and therefore their dietary diversity and food security). Kiango Singoti, Bombani village said: “Because different types of fruits need different types of fields, it has forced me to cultivate multiple plots [in multiple locations]”. Similarly, Benjamin Njiku explained: “I plant some trees and some other food crops so it is like a forest but not an ordinary forest. Because maybe this area once had trees, but not when I came, so I decided to plant sugarcane (*Saccharum* spp.) and it helps me. But I have planted trees in other areas… in other areas I plant food crops which do not resemble forest.”

The few participants who did not link diet and dietary diversity to agriculture and agrobiodiversity were among the most disadvantaged of the respondents; they also struggled to articulate all aspects of their life, diet, nutrition and health. For example, Tabea and Dominic John of Misalai village were not cultivating their farm: they explained it was “too difficult”, although both appeared to be young and healthy (as an explanation, Dominic said “it’s easier to plant after hoeing (tilling), but hoeing is what impedes me”). They were living almost entirely off the small earnings Tabea made from the restaurant attached to their house where she sold tea and *mandazi* (fried sweet dough) and very small amounts of cash from Dominic’s occasional business endeavours. While it was unclear why exactly the family was so adverse to farming (perhaps social or psychological reasons), it was clear that there were obstacles other than physical ability preventing them from farming (Tabea regularly carried very heavy loads of firewood needed cook *mandazi*): emphasizing the benefits of farming would have just made them feel worse about the fact that they were not engaged in farming. Likewise, Mary Mathayo (a single mother, the poorest of the 15 case study households, in Kiwanda village) did not discuss a link between agriculture and dietary diversity. She cultivated only maize and cassava and lived off the sale of sugarcane alcohol. The concerns and efforts of both these households were focused on small business enterprises, which produced small amounts of cash with which they purchased very basic food items.

#### Spatial and temporal availability of diverse foods

Availability of different foods (both seasonally and geographically) in general was reported as a limitation to dietary diversity; some foods simply were not available in some places or at some times. Even if one has enough money, if a food item is not available, it cannot contribute to dietary diversity. However, seasonal variation is perceived to affect long-term dietary diversity. Additionally, the varying availability of foods from the farm can be mitigated by purchasing foods when they are not available from the farm. For example, Rehema Amiri a single mother and successful business-woman in Shambangeda village explained:

“I have avocado (*Persea americana* Mill.), guavas (*Psidium guajava* L.) and pineapples (*Ananas comosus* L), everything is there [in the farm]. There are many guavas, we just pick them, and the children eat them.... They [fruits] go with their own seasons. There are seasons you will get many fruits, and then there are many seasons when you will get a few fruits. In that season when fruit are scarce you will buy a few, like pineapple that is normally available in the field, when it is not there, before it is ripe [you must buy it] or oranges (*Citrus sinensis* L.) and bring them home.”

#### Income, cash availability and socio-economic status

Wealth or income was reported as an important determinant of diet and dietary diversity. It was perceived to affect dietary diversity both directly (through purchasing power), as well as indirectly by modifying agrobiodiversity: wealthier people could afford to purchase more types of seeds and other agricultural inputs, could afford to hire help with agricultural labour, and usually had access to more land, all of which increase their ability to grow a greater variety of crops. Both wealthy and poor participants identified wealth or poverty as a factor limiting some people’s access to food and dietary diversity. For example, Tumaini and Kibua Daudi (recently returned to the area, making a successful living by small business, Bombani village) and Anna and Ernest Singano (poor, renting their home from the tea plantation for which Ernest works, farming with the goal of leaving the tea estate, Shambangeda village) talked about their lack of access to land/land tenure affecting their agrobiodiversity, food security and dietary diversity. Trade-offs between obtaining food by way of agriculture and by purchasing it were frequently discussed. While lack of money could be made up for by successful agricultural endeavours, wealth gave a household choice and lack of wealth required the household to balance more activities. Local people not only drew connections between greater wealth and higher agrobiodiversity supporting high dietary diversity, they also identified lack of wealth and lack of crop diversity as decreasing their ability to maintain their dietary diversity.

#### Livelihood diversity

In many cases, livelihood diversity was seen to support dietary diversity in the absence of wealth or agrobiodiversity. Certain livelihood activities, such as livestock keeping, consistently emerged as beneficial for dietary diversity. The family of Ramadhani Juma had excellent dietary diversity, which he attributed to livelihood diversity. He discussed how his black pepper (*Piper nigrum* L.) harvest gave him lump sum earnings once a year, how bananas could be sold throughout the year (although only for a small amount of money), how he grew maize and beans for home consumption, and how his dairy cows helped him pay for his children’s school fees. He took occasional work as a mason, as well as tailoring work at holidays, and his wife had a small business selling fried fish. Rehema Amiri, a single mother, farmer and business owner in Shambangeda also talked about how her efforts in both business and agriculture helped her to ensure her family’s well-being, nutrition and dietary diversity.

Indeed, a lack of livelihood diversity was observed in case study households with some of the lowest dietary diversity. Participants noted that households that focus all their agricultural efforts on cash crops were more likely to encounter difficulties maintaining a good diet. A number of participants noted that those who worked as labourers on the tea estates were significantly disadvantaged and had very monotonous diets if they didn’t have any other livelihood activities. Anna Ernest, who lived on the tea estate where her husband worked in Shambangeda village commented: “If I plant cassava like this one, I do not need to buy it, even beans. You will find them [those who work for the tea company and don’t engage in agriculture] drinking tea alone, or tea and boiled banana. And as for cultivated vegetables, I will harvest leafy vegetables and they will eat only *dagaa*.”

However, in a number of cases, certain livelihood activities actually acted to decrease diet quality and dietary diversity. In one (Tabea and Dominic John) of the two households where the mother of the home ran a small restaurant and prepared *mandazi* (fried sweet dough), the dietary diversity was quite low. *Mandazi* preparation is very time consuming, leaving the mother with limited time to cook other meals, as well as less impetus to cook, as the family can fill up on *mandazi.* Musa Mbwana, the head of another household, was a well-known traditional healer. His family consumed chicken frequently, as chickens are brought by patients for sacrifice during treatment (and the meat is given to the healer as part of the payment for his services). In his household, the ready access to chicken meat seemed to act to decrease the consumption of other side dishes (especially vegetables), and thus the dietary diversity of the family.

### Other determinants of dietary diversity

Other less prominent determinants of dietary diversity included: large household size, gender, and personality and family traditions.

#### Household size

A number of households identified large family size as an obstacle affecting diet and food security. Although a larger family size increases the demands on the adults in the household and decreases their ability to overcome obstacles that require monetary input, in some cases it may increase dietary diversity. This is because it is more difficult to get enough of any one type of food to feed a large family, and less likely that there will be leftovers to eat at the next meal.

#### Gender

For female-headed households (single father headed households were extremely rare), a limited work force is often a major limitation. Mary Mathayo, a single-mother and the most disadvantaged of the case study households noted:

“I was married and I separated. Now, I earn my living by *sukuma miwa* (literary means pushing sugarcane, refers to making and selling sugarcane alcohol)… Other households eat better than mine because my strength is that of only one person, *kwasabu mkono wangu ni mmoja* (because I have one pair of hands, i.e. she is a single parent).”

However, the role of gender in determining dietary diversity in the East Usambaras is complex; gender often affects other factors that determine dietary diversity. For example, traditional land inheritance laws which disadvantage women can limit agriculture and crop diversity. In one household, because the wife, from the area, had married a man from the West Usambara Mountains, her brothers barred her from inheriting or even using her family’s land after her father’s death. In another family, patrilineal land tenure practices limited the type of crops the wife could plant on her husband’s land (especially because she had sons from another marriage): permanent crops are seen as an assertion of tenure. It is important to note that there were many success stories of women overcoming gender-based obstacles; in fact, many of the most successful (in terms of diet, dietary diversity and agriculture) households were run by women.

#### Personality and tradition

A number of personal characteristics were also common themes in discussions of what affects dietary diversity and nutrition. Personality emerged particularly when people with better diets/higher dietary diversity (especially poorer households that still managed to maintain good dietary diversity) tried to explain why other households might not have the same quality and diversity in their diets. Some participants simply said that people “don’t like to/don’t want to” pursue various activities needed to ensure dietary diversity. “Each person has their own thoughts or ideas or plans” was another very common explanation. An individual’s knowledge, determination, drive, dedication, effort and motivation were often cited as aspects of personality, which can support improved dietary diversity. For example: “You can get many types of vegetables, but it all depends on the effort/determination (*juhudi*) of the mother of the house… to struggle to find them. Because there are many mothers who don’t want to go to the bush to look for vegetables, they get money and buy *dagaa*. Others are determined to look for vegetables.” explained Saidi Kombo.... “[I am more able/determined than other women] because I really like leafy vegetables.” continued Amina, his wife.

Other participants linked a person’s choices and habits to their family’s traditions and commitment to culturally held food preferences and taboos. While in some settings, cultural taboos are universally held and adhered to, in the East Usambara Mountains many food taboos varied from one family to another. Only a few taboos, like those against eating snails and monkeys, were held by the majority of people.

Rarely did people say that others lacked dietary diversity because they were in some way poorer or disadvantaged, suggesting that dietary diversity was perceived as something all people in their community (rich and poor) can achieve.

## Discussion

These data demonstrate that dietary diversity is perceived as important for appetite and overall food consumption. There was a high degree of agreement among participants from a range of backgrounds about the concept of dietary diversity and its benefits for enhancing appetite. These findings suggest that the concept is a salient part of local nutrition knowledge. These results also contribute to the growing understanding of how dietary diversity is associated with biodiversity. Agriculture, agrobiodiversity and landscape heterogeneity, along with wealth were some of the most commonly reported determinants of dietary diversity. Household size, livelihood diversity, and gender were also perceived to affect dietary diversity.

**Fig. 1:**
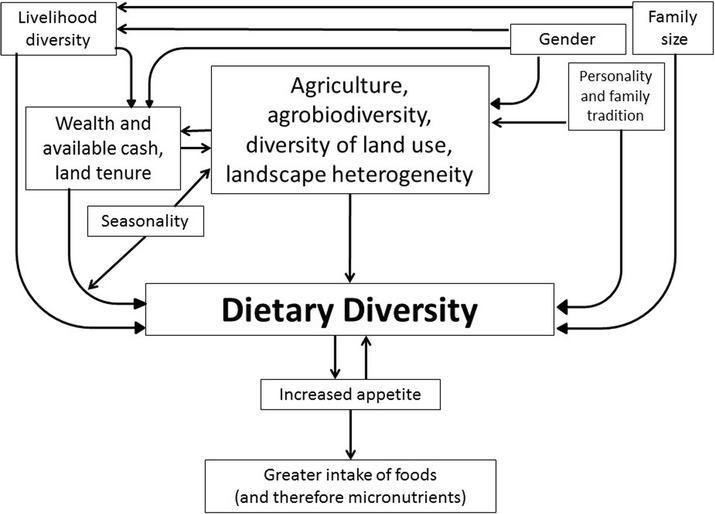
Diagram of the most salient relationships between determinants and outcomes of dietary diversity, showing an interpretation of how they interact within the social-ecological system (*arrows* indicate associations that can be either positive or negative, they are not meant to indicate causation)

Figure 1 represent our effort to visualize the reported determinants of dietary diversity and their interactions. The figure highlights our interpretation, based on the results presented herein and our wider understanding of the field site, of how the drivers of dietary diversity are situated and interact within the larger complex social-ecological system. To our knowledge this is the first study that describes the importance of landscape heterogeneity for dietary diversity, a factor that has never previously been described as a potential driver of dietary diversity and should be further investigated.

Many of the relationships between dietary diversity and health, and dietary diversity and environment reported by local people in this study parallel those reported in other studies, both quantitative and qualitative (including the quantitative results from the same research project). Table 1 summarizes relationships between dietary diversity and health from the general scientific literature, quantitative research from the same communities included in this study, alongside a summary of the local knowledge presented herein. The same three sources of information on the drivers of dietary diversity are also presented.

**Table 1:** Summary of knowledge about the importance and drivers of dietary diversity (DD) from three different sources (local knowledge, scientific knowledge/quantitative results from same study, scientific knowledge/quantitative results from other studies)

Scientific literature	Quantitative data from the study communities	Qualitative data from local people in this study
Importance of Dietary Diversity		
• DD associated with overall food consumption, energy intake and satiety [51, 64–69] • DD associated with dietary quality, nutrient intake, nutrient density (likely explaining links to child growth and other anthropometric and biochemical markers of nutrition) [68–71]	• DD associated with overall energy intake [46] • DD associated with intake of most nutrients and nutrient adequacy (Mean Adequacy Ratio, MAR) [46] • After controlling for energy intake, DD no longer associated with intake of most nutrients [46]	• DD important for appetite and enjoyment of food • DD important because “each food has its own value”
Drivers of Dietary Diversity		
• DD linked to agrobiodiversity in at least seven studies [46, 52, 72–78], • DD associated with forest cover in three studies [47, 52, 79, 80]. • DD linked to vegetable production [81] • DD linked to home gardens [52, 82] • Season may increase/decrease DD [83, 84] • Wild food use associated with higher DD [54] • DD also associated with wealth, household size, education and other economic and demographic variables	• DD associated with agrobiodiversity (crop diversity) [46] • DD associated with forest cover [46, 47]. • No differences in DD between wet and dry season, but difference in sources of foods [46, 48] • DD associated with wealth and market access but not with sex or education of the head of the household (unpublished data from the study) [46]	• Agriculture, agrobiodiversity • Land use diversity/landscape heterogeneity • Different foods eaten seasonally • Wealth, available cash, land tenure • Livelihood diversity • Family size • Gender • Personality, family tradition, taboos

Local knowledge about the importance of dietary diversity recorded is well-aligned with scientific findings: participants’ emphasis of the role of dietary diversity for appetite, over its importance for vitamin and mineral intake or other nutrition-related health outcomes, was likely due to the fact that vitamins and minerals are concepts that are not well integrated into existing local knowledge systems. Older nutrition and nutritional anthropology research has similarly reported that diets that lack diversity induce boredom and undereating, especially in children [51]. The fact that many participants talked about dietary diversity and having (enough) food in general somewhat interchangeably, suggests not only that food security is important to people, but that it is associated with dietary diversity (as in the scientific literature).

Scientific evidence of the drivers of dietary diversity vary between sites and studies (first column Table 1). Local knowledge in this study suggests that land use diversity and landscape heterogeneity are important for dietary diversity; factors which have not, to date, ever been examined using dietary survey data [52]. Local knowledge that crop diversity is important for food security and resilience has been previously described: farmers around the world report that crop diversity and agrobiodiversity provide them with security in the face of environmental, climate, economic and social change (e.g., in Nepal [53]). One recent research project has also looked at local knowledge of the importance of agrobiodiversity in Rwanda, with remarkably similar results to this study [37]. The research in Rwanda reported that local people identified dietary diversity as one of the two most important reasons they valued agrobiodiversity (along with income generation), and dietary diversity was perceived as important for health and nutrition: “farmers said they liked to grow diverse crops because they wanted to eat different foods, that eating the same food caused health problems, and diverse foods are important for nutrition” [37]. For example, one of the farmers interviewed by Isaac et al. [37] stated: ‘If you have production from all of these [crops] there’s no hunger. And to change the food – today if we eat squash and beans the next day we can eat something else…Life is strong – there’s no disease in the body and children don’t get sick because they change the food every day’ [37].

Interestingly, despite wild food being widely consumed in the study site [48], and having been shown to be associated with greater dietary diversity [54], there was limited mention of wild foods by local people in their discussion of the drivers of dietary diversity.

Discrepancies between scientific knowledge and local knowledge of dietary diversity presented herein should not in any way reduce the validity of local knowledge. The assessment of local knowledge against “scientific truths” perpetuates a dichotomy, in which local knowledge is qualified relative to a ‘superior’ knowledge; dichotomies which maintain colonial cultural supremacies and perpetuate hegemony [55, 56]. In this case, the discrepancies between local knowledge and the other research findings from this study could easily be a result of the well-known imperfections in dietary data collection tools [46]. In fact, this qualitative research may have identified drivers of dietary diversity that have simply not been identified or tested yet using quantitative methods (e.g., land-use diversity).

At the same time, we acknowledge the subjective nature of qualitative analysis, the possibility that other themes/categories could be apparent to other researchers or that the relative importance of themes/categories could be judged differently. The fact that the first author introduced herself as a nutrition researcher, could have led participants to give responses which they perceived to align with the scientific knowledge system. However, she was able to elicit a diverse range of responses from local people, including many that were not close to scientific concepts (and was able to do this when Tanzanian members of the team with university degrees in science often were not able to) suggesting that this aspect of researcher bias was largely overcome.

The fact that “personality” emerged as an explanation given as to why some members of the community were unable to maintain a diverse diet highlights one of the limitations of drawing on local perceptions of determinants of health: poor people are often blamed for making bad decisions, or being lazy, when in reality there are structural barriers, including social, cultural and environmental barriers, that prevent them from making healthy choices [57, 58].

In the face of changing food systems and dietary patterns [13], public health nutrition policy and programs will need to find messages that support cultural dietary traditions, promote healthy dietary behaviour and are easily integrated into existing knowledge systems. The Tanzanian primary school curriculum has been an efficient means for promoting healthy diets in this site. Many of the concepts seen in this research also seen in the primary school science curriculum and participants reported school as a source of information. Compared to scientific approaches to many health issues (which often focus on causes and treatment of disease or deficiency), scientific discourse about dietary diversity focus more on health (and how to maintain it) [55]. Similarly, the local knowledge examined in this research tended to focus on the health-giving components of diet and food. Compared to other forms of nutrition knowledge which are often heavily nutritionalized and medicalized [59], dietary diversity presents an ideal starting place and foundation for education about healthy diets.

## Conclusions

Shell-Duncan and McDade [60] highlight the importance of ethnographic data on health and nutrition knowledge for interpretation of nutrition survey results: they describe higher rates of inadequate iron intake among girls than boys in a Rendille community in northern Kenya and link this to cultural classifications of ‘soft’ foods (including rice, maize porridge, and tea), important for girls, and ‘hard’ foods (including meat, blood, and beans) important for boys. Worsley [34] points out that “…‘messages’ are often accepted or rejected according to their consonance with prior beliefs”. The gap between scientific and local knowledge systems remains a major, and rarely addressed, issue in nutrition interventions with education or behaviour change components. Unlike Shell-Duncan and McDade [60], which describes local nutrition knowledge that is quite different from scientific knowledge, in this study, local knowledge of dietary diversity was well aligned with scientific knowledge.

The universal use of the Swahili language and primary school education are legacies of Tanzania’s socialist era [61, 62], and have likely been a driving force behind the creation of consensus among local people on nutrition knowledge. These are strengths that future public health policy and programs should seek to build on. There is room to incorporate ethnonutritional concepts in the National Primary School science curriculum, and to adapt the curriculum to local contexts. In the face of changing dietary patterns and nutrition burdens across Africa, attention to the diet and nutrition information in school curriculums should be prioritized.

The qualitative approach to ethnonutrition used in this research has revealed that local people perceive a strong link between agriculture and agrobiodiversity (and by extension environmental health in general) and human diet and nutrition. Landscape heterogeneity was reported as important for dietary diversity. Maintenance of agriculture when households shift to other sources of income was also reported as important. Crop varietal diversity was reported as important for maintaining dietary diversity across seasons. Income, livelihood diversity and household size were also reported as important. Nutrition research has struggled to accept other ways of knowing as equal to knowledge generated through scientific enquiry. New paradigms are needed to achieve greater and more efficient knowledge communication in nutrition interventions and public health nutrition. Overlaps between scientific and local knowledge systems (such as dietary diversity) offer an excellent platform to provide novel health and nutrition information to local communities; such an approach should enable novel information to be more readily integrated into existing local knowledge systems.

## Additional files

Additional file 1: Info Brief: Diet and Nutrition in the East Usambaras (in Swahili). (PDF 242 kb)

Additional file 2: Info Brief: Nutrition and Environment in the East Usambaras (in Swahili). (PDF 567 kb)

## Footnotes

1 Definitions of agrobiodiversity vary; the Convention on Biological Diversity (CBD) defines it as all living organisms associated with agriculture, crops and livestock and the ecosystem of which they are a part, while others, including (Bioversity International) it includes only cultivated species, herein we use crop diversity to specify and agrobiodiversity to refer to both the more specific and the more general definitions combined. It is important to note that plant taxonomy in local knowledge systems often differs from scientific classifications [63]. In this work when “type” in “type of food” “type of vegetable” or “type of fruit” was discussed with local people, the definition of “type” was left to the informant’s discretion. Generally “type” used by participants matches traditional agricultural classification of crops, and does not refer to variety unless specified.
