# Supplementary material for: The determinants of dietary diversity and nutrition: ethnonutrition knowledge of local people in the East Usambara Mountains, Tanzania
Source: J Ethnobiol Ethnomed. 2017 Apr 27;13:23. doi: 10.1186/s13002-017-0150-2 (PMC5406938; doi:10.1186/s13002-017-0150-2)
Supplement: Supplementary file 1 — Info Brief: Diet and Nutrition in the East Usambaras (in Swahili). (PDF 242 kb) [file 13002_2017_150_MOESM1_ESM.pdf]

# - Info Brief -

Nutrition and Environment in the East Usambara Mountains

Methodology:
Data collected in 2009 in 6 villages (Bombani, Tongwe, Kiwanda, Kwatango, Shambangeda, Misalai) in N=274 households (mothers and children aged 2- 5 years old).

Wild foods:

- 92 wild food species (vegetables, fruit, meat/fish, mushrooms)
- 62% obtained from the farm, 21% purchased and only 12% from the forest
- Consumed by 98% of people in wet season and 93% in the dry
- Contributing 15.4% of items in the diet in the wet and 8.9% in the dry season from wild species
- Wild foods contributed 2% of total energy, 31% of vitamin A (RAE), 20% of vitamin C, 19% of iron , 16% of calcium and 12% of folate (vitamin B) in the diet
- People who spent more time farming ate more wild foods

"*Those leafy vegetables are in the farm and if today I do not have money it will force me to leave home and waste time and go to look for that vegetable so that it can fill that gap*." Beatrice Akida

Agriculture:
- 41% of food items from farm, percent of diet obtained on farm was positively correlated with overall diet quality (MAR)
- Agricultural diversity (crop diversity) was positively associated with dietary diversity
- Agricultural diversity (crop diversity) was positively correlated with children's over all dietary adequacy (MAR)

Water:
- Access to clean drinking water (from a tap) was associated with children's growth, indicating that access to clean drinking water likely helps to reduce infection rates (from worms and other parasites)

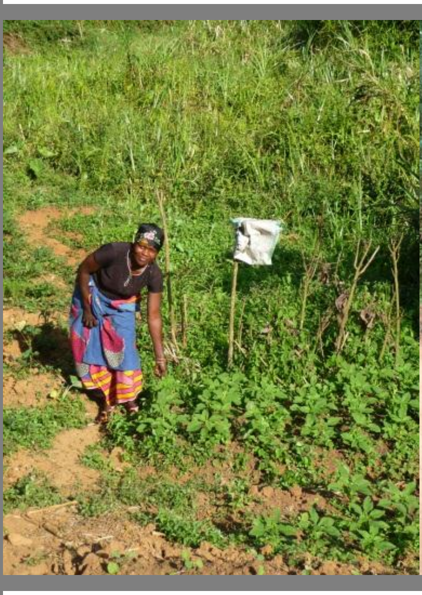

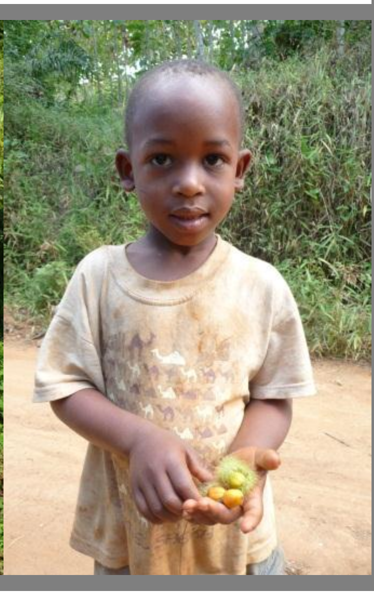

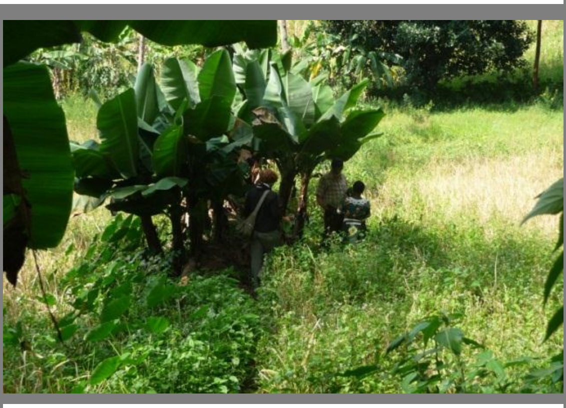

Trees and Forests:

- Children living closer to forest / with more trees near their house had higher dietary diversity (ate more different types of foods)
- Children who ate food from forest had better diet (higher dietary diversity, higher nutrient density)

Tree cover and vegetables:
- Vegetable consumption associated with better quality diet and consumption of micronutrients
- Vegetable consumption much lower in the in the dry season than the wet season (21% vs. 43% of children had consumed vegetables the previous day)
- In the dry season, but not the wet, those who had consumed vegetables had greater tree cover in close proximity to their home (604ha vs. 440ha for children who had and had not consumed TVs, p<0.01 in a Mann-Whitney U test).

Roads:
- Children's overall nutrient adequacy (MAR) and nutrient density were lower in villages with better road access
- Use of purchased food was higher in villages with greater road access. Use of purchased food was highly negatively correlated with overall nutrient adequacy (MAR)
- Roads are important for access to medical care and markets for agricultural products (but these topics were not included in this research)
- As communities gain better road access they should avoid increased purchased food consumption and maintain agricultural activities (at least a garden or small field)

Research conducted in collaboration with McGill University, Montreal Canada; Bioversity International, Nairobi, Kenya and the World Vegetable Centre, Regional Centre for Africa, Arusha, Tanzania. Research funded by IDRC, Canada
Results from Bronwen Powell's PhD Research(bronwen.powell@hotmail.ca)
