# Supplementary material for: The determinants of dietary diversity and nutrition: ethnonutrition knowledge of local people in the East Usambara Mountains, Tanzania
Source: J Ethnobiol Ethnomed. 2017 Apr 27;13:23. doi: 10.1186/s13002-017-0150-2 (PMC5406938; doi:10.1186/s13002-017-0150-2)
Supplement: Supplementary file 2 — Info Brief: Nutrition and Environment in the East Usambaras (in Swahili). (PDF 567 kb) [file 13002_2017_150_MOESM2_ESM.pdf]

# - Info Brief -

Mlo na Lishe Bora kwa Milima ya Usambara Mashariki

Mbinu: Takwimu zilizokusanywa mwaka 2009 katika vijiji 6 (Bombani, Tongwe, Kiwanda, Kwatango, Shambangeda, Misalai) kutoka kaya N = 274 (mama na watoto wenye umri wa miaka 2-5)

Virutubishi ulaji: Virutubishi vyenye uwezekano wa kuwa upungufu (chini mno), kipimwa kama vyenye uwiano wa chini kukidhi mahitaji kwa (nutrient adequacy ratios (NARs) – ulaji wa watoto / USDA mapendekezo kwa umri)

Virutubishi vyenye uwezekano mkubwa zaidi kuwa pungufu katika mlo wa watoto (kwa mpangilio umuhimu):

- Zinc
- Vitamini B_{12}
- Vitamini A
- Calcium
- Chuma

Ukuaji wa Watoto: Kiwaneo cha kudumaa ('Stunting' = urefu-kwa-umri Z score (HAZ) <-2 SD, inajihihirisha kama ukuaji usioridhisha ilinganishwa na viwango vya kimataifa (WHO) vya ukuaji curves).

- Wastani HAZ = -1.73 ± 1.23
- 39% ya watoto wamedumaa
- Watoto wanaotuma maji ya bomba walikuwa na uwezekano mdogo wa kudumaa kuwa ikilinganishwa na wanaotumia maji ya chemchemi/ mtoni (31% vs. 58% kudumaa, p=0.001)
- Hakuna tofauti kati ya wavulana na wasichana
- Vipimo vya ubora wa mlo na lishe bora havikuwa uhusiano kitakwimu na ukuaji wa watoto

Kubadilisha Mlo: Ufahamu wa asilia unaonyesha kuwa kubadilisha mlo ni muhimu katika kuhakikisha hamu ya chakula, hasa kwa watoto.

Matokeo ya utafiti yanaonesh kwamba ubadiisha wa milo kwa watoto (FVS, DDS_{14} over 1 day and 7 days and DDS_6 over 1 day) inawiana na ulaji wa virutubishi kwa kiwaneo kikubwa zaidi

FVS: Nambari ya ainia ya vyakula
DDS_6: Nambari ya makundi ya vyakula, katika 6
DDS_{14}: Nambari ya makundi ya vyakula, katika 14

Viashiria vya Ubora ujumla wa Mlo:
Ubora ujumla wa mlo kipimo kwa MAR (mean adequacy ratio, wastani ya NARs ya virutubishi 11)

Ubora ujumla wa mlo (MAR) juu ilikuwa uhusiano na:

- Kukula mboga mboga zaidi
- Kukula nyama, kuku na samaki bichi zaidi
- Kukula chini ya vyakula kununuliwa
- Kubadilisha mlo zaidi

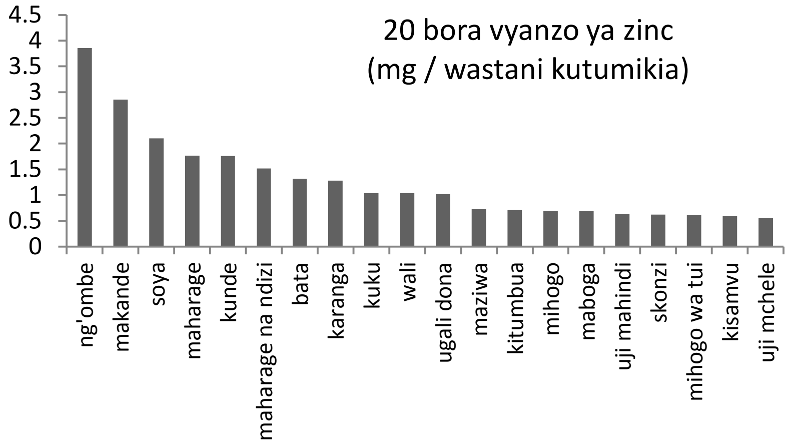

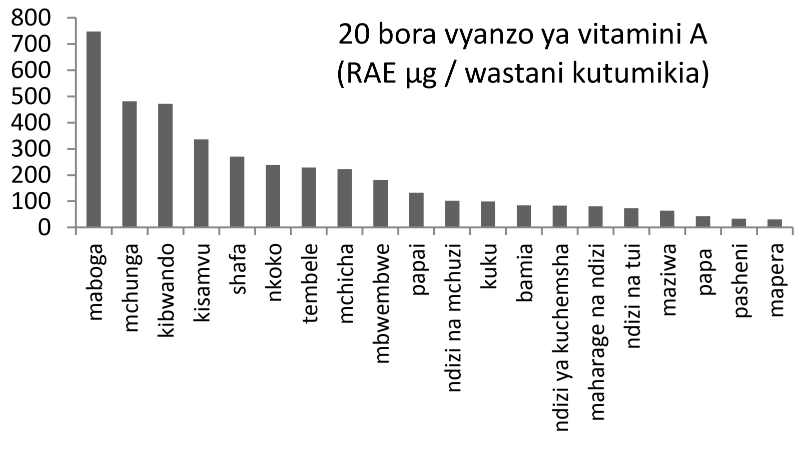

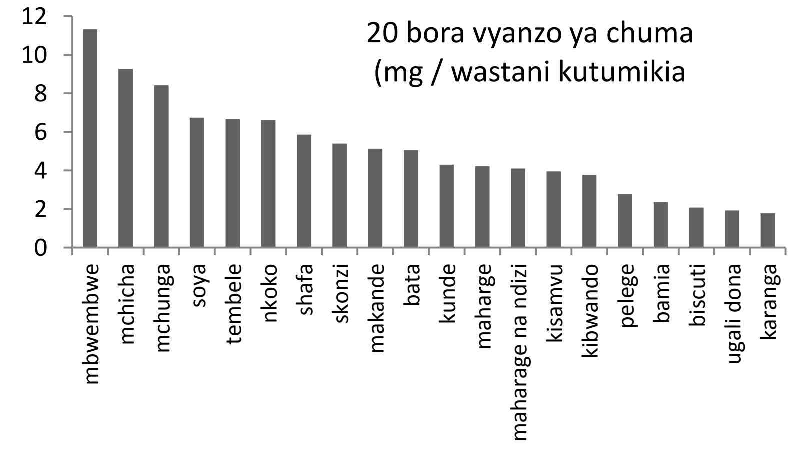

Utafiti kufanya kwa kushirikiana na McGill University, Montreal, Canada; Bioversity International, Nairobi, Kenya; na the World Vegetable Centre, Regional Centre for Africa, Arusha, Tanzania. Research kufadhiliwa na IDRC, Canada

Matokeo ya utafiti ya PhD ya Bronwen Powell(bronwen.powell@hotmail.ca)
